# CoQ10 enhances the efficacy of airway basal stem cell transplantation on bleomycin-induced idiopathic pulmonary fibrosis in mice

**DOI:** 10.1186/s12931-022-01964-4

**Published:** 2022-02-26

**Authors:** Huanbin Liu, Shuna Liu, Jinjun Jiang, Yidi Zhang, Yulong Luo, Jingxin Zhao, Jian Xu, Yuan Xie, Weiping Liao, Wei Wang, Yichu Nie, Shiyue Li, Wenbin Deng

**Affiliations:** 1 School of Pharmaceutical Sciences (Shenzhen), Sun Yat-Sen University, Guangzhou, 510275 People’s Republic of China; 2 State Key Laboratory of Respiratory Diseases, The First Affiliated Hospital of Guangzhou Medical University, Guangzhou Medical University, Guangzhou, 510120 People’s Republic of China; 3 Foshan Fourth People’s Hospital, Foshan, 528000 People’s Republic of China; 4 Clinical Research Institute, The First People’s Hospital of Foshan & Sun Yat-Sen University Foshan Hospital, Foshan, 528000 People’s Republic of China

**Keywords:** Idiopathic pulmonary fibrosis (IPF), Airway basal stem cells (BCs), Coenzyme Q10 (CoQ10), Cell transplantation

## Abstract

**Background:**

Recent studies have demonstrated that airway basal stem cells (BCs) transplantation can ameliorate bleomycin-induced idiopathic pulmonary fibrosis (IPF) through lung regeneration promotion. However, BCs under oxidative stress in the alveolar microenvironment are poor in survival, causing unsatisfied efficacy of BCs transplantation. In this study, we investigated whether Coenzyme Q10(CoQ10) counteracts oxidative stress in the alveolar microenvironment, thus improved the efficacy of BCs transplantation for IPF treatment.

**Methods:**

The protective effects of CoQ10 on H_2_O_2_-induced BCs apoptosis and cytoplasmic reactive oxygen species (ROS) level were tested by flow cytometry in vitro. The therapeutic effects of BCs combined with CoQ10 were compared to a single BCs transplantation protocol in IPF treatment after 2 weeks and were evaluated by parameters including changes of body weight and survival rate, as well as various levels of pulmonary inflammation, α-SMA expression and hydroxyproline (HYP) in IPF mouse lung tissues.

**Results:**

CoQ10 preincubation with BCs (10 mM, 24 h) significantly reduced the late apoptosis of BCs and the number of oxidative stressful BCs as a result of H_2_O_2_ stimulation (1 mM, 6 h) in vitro. IPF mouse model was constructed through bleomycin (5 mg/kg) intratracheal instillation. Bleomycin-induced IPF mice showed weight loss continuously and mortality increased progressively during modeling. Serious pulmonary inflammatory cell infiltration, collagen fiber proliferation, and collagen protein deposition were observed in lung tissues of IPF mice. Though BCs transplantation alone improved indicators above in bleomycin-induced IPF mice to some extent, the combination with CoQ10 improved the transplantation efficacy and obtained better therapeutic effects.

**Conclusion:**

CoQ10 blocked H_2_O_2_-induced apoptosis of BCs and ROS production in vitro, and enhanced the efficacy of BCs transplantation against bleomycin-induced IPF in mice.

## Background

Idiopathic pulmonary fibrosis (IPF), regarded as a “tumor-like” disease, is caused by lung epithelial cells injury and repair disorder. IPF is more common in the elderly, with a median survival time of nearly 3 years. With the global aging population, the incidence of IPF is increasing yearly. At present, only Pirfenidone and Nintedanib are recommended as first-line therapeutic drugs in IPF treatment. Though they can postpone the IPF disease progress, they cannot realize the radical cure of IPF [1–4]. Recent researches suggest that stem cell transplantation may provide a potential treatment [5–7].

Stem cells hold differentiational potentials and can be differentiated into alveolar epithelial cells to repair the damage and fundamentally delay or reverse IPF. Therefore, stem cell transplantation, such as mesenchymal stem cells (MSCs) [8–13] and induced pluripotent stem cells (iPSCs) [14–18], were widely used for allotransplantation to repair injury of IPF. Studies have shown that transplanting bone marrow-derived MSCs significantly ameliorated lung injury and pulmonary fibrosis in bleomycin-induced IPF animal models [9] and the safety of transplanting human MSCs in patients with IPF has also been verified in clinical trials [10]. Studies have shown that MSCs have the homing function. After lung injury, bone marrow-derived MSCs migrate and home to the lungs, decrease lung inflammation and collagen deposition in the bleomycin-induced pulmonary fibrosis animal [8, 11]. Many viewpoints also demonstrated that MSCs play an anti-fibrosis role through paracrine and immune regulation [12, 13]. Although injection of MSCs can alleviate the accumulation of fibers in lung tissue and lung injury induced by bleomycin, most cells transplantation researches carried out in the early inflammation stages instead of in the late fibrosis stage. Moreover, the main questions come from the tumorigenicity and multi-directional differentiation potential of MSCs, which means MSCs may not differentiate into type II alveolar epithelial cells, and further promote the regeneration of injured lung epithelial cells. iPSCs have been reported to differentiate into alveolar epithelial cells in vitro [14–18], however, the tumorigenesis and the ability of iPSCs to differentiate into alveolar epithelial cells in vivo have yet to be verified.

Airway basal stem cells (BCs) are a kind of adult stem cells located in the basal layer of the airway and can self-proliferate and renew [19]. Its specific makers include KRT5 and P63 [20]. Several studies have demonstrated that airway basal stem cells have potent regenerative capacity after lung injury [21], and BCs transplantation can ameliorate pulmonary fibrosis [22]. However, cell survival is affected by the microenvironment during transplantation, and the efficacy of cell transplantation is a standing challenge. Coenzyme Q10 (CoQ10) or ubiquinone (2,3-dimethoxy-5-methyl-6-polyprenyl-1,4-benzoquinone) is a lipophilic molecule found in the phospholipid bilayer of cellular membranes and is concentrated in the mitochondrial inner membrane. As a mitochondrial electron transport chain component, CoQ10 can make mitochondrial mass, improve mitochondrial function, and inhibit ROS generation [23, 24]. It has been shown that CoQ10 supplementation can reduce DNA double strand damage and increase the life cycle of peripheral blood monocytes [25]. CoQ10 could inhibit d-gal-induced cell aging [26] and protect neural stem cells against hypoxia by enhancing survival signals [27].

At present, no literature report combining with CoQ10 can improve the survival efficiency of transplanted stem cells. The purpose of this study was to investigate the effect of BCs transplantation combining with CoQ10 on bleomycin induced pulmonary fibrosis mice.

## Methods

### Chemicals and reagents

Bleomycin was purchased from Hanhui Pharma Co., Ltd. (Hangzhou, China). 4% paraformaldehyde was purchased from Wuhan Saiweier Biological Technology Co., Ltd (Wuhan, China). Hydroxyproline assay kit was purchased from Nanjing Jiancheng Bioengineering Institute (Nanjing, China). CoQ10 (purity > 98%, using HPLC) was purchased from Sigma-Aldrich (St. Louis, MO, USA), dissolved in DMSO (St. Louis, MO, USA), and diluted into the culture medium. The final concentration of the DMSO was 0.1%. Annexin V-FITC apoptosis detection kit and total ROS/superoxide detection kit were purchased from Dalian Meilun Biotechnology Co., Ltd. (Dalian, China).

### Cell culture

BCs were obtained from the First Affiliated Hospital of Guangzhou Medical University (Guangzhou, China), which were primarily isolated and cultured from airway mucosal biopsy samples from non-fibrotic patients (adult male, diagnosed with chronic cough) undergoing fiberoptic bronchoscopy based on previously published method [28, 29]. BCs were cultured in DMEM/F12 medium containing 5 μM ROCK inhibitor Y-27632 (Sigma, Cat. SCM075), 1 μM A-83-01 (Sigma, Cat.SML0788), 1 μM DMH-1 (MedChemExpress, Cat. HY-12273), 1 μM CHIR99021 (Sigma, Cat.SML1046), and 1× Antibiotic–Antimycotic medium (ThermoFisher, Cat. 15240062) at 37 °C with 5% CO_2_ in a humidified atmosphere. The culture medium was changed every 3–4 days. Cells with 80% confluency were passaged and the generation between P2 to P8 were used for the experiment. MSCs were obtained from Southern Medical University (Guangzhou, China).

### Cell groups and treatments

Groupings: (1) blank group: human BCs were cultured in medium without CoQ10 and H_2_O_2_ before measurement, and the other steps were the same; (2) H_2_O_2_ group: BCs were cultured with 1 mM H_2_O_2_ for 6 h before measurement; (3) CoQ10 + H_2_O_2_ group: BCs were incubated with 10 mM CoQ10 for 24 h, and then changed into medium with 1 mM H_2_O_2_ for 6 h before measurement.

### Determination of intracellular ROS levels

ROS assay kit (DCFH-DA, MedChemExpress, Cat. HY-D0940) was used to detect the level of reactive oxygen species in human BCs of each group. Cells were treated by 10 mM CoQ10 and/or 1 mM H_2_O_2_ as the preceding steps, followed by PBS wash to remove the residual H_2_O_2_. Each cell was then added in 100 μL serum-free medium containing 2 μL of 5 mM DCFH-DA, incubate the microplate for 30 min at 36 °C in the dark with occasional shaking. Then carefully discard the liquid within each well and wash the cells twice with PBS. The ROS concentration in each well was detected by flow cytometry (BD Biosciences, NJ) or Nikon microscope (Tokyo, Japan).

### Determination of cells apoptosis

After pretreatment by CoQ10 and H_2_O_2_, BCs were harvested through trypsinization and washed with cold PBS. BCs were resuspended by PBS and centrifuged at 3000 r/min for 5 min, then the supernatant was discarded and the pellet was resuspended in 1× binding buffer at a density of 1.0 × 10^6^ cells/mL. 100 μL of the sample solution was transferred to a 5 mL culture tube, and incubated in the photophobic environment with 5 μL of FITC-conjugated annexin V (Pharmingen) and 10 μL of PI for 15 min and resuspended every 3 min. 400 μL of 1× binding buffer was added to each sample tube, and the samples were detected by flow cytometry within 1 h.

### Animals

Fifty male C57/B6 mice (weighing between 20 and 30 g) were purchased from Laboratory Animal Center of Sun Yat-sen University (SYXK 2016-0112). The animals were acclimated to the laboratory for at least 7 days before experiments. The animal care and use complied with the Provisions and General Recommendation of the Chinese Experimental Animals Administration Legislation. Animal experiment protocol was approved by Laboratory Animal Center of Sun Yat-sen University.

### Animals’ groupings and treatments

Mice were randomly divided into five groups (n = 10): blank group, model group, BCs group, MSCs group, and CoQ10 + BCs group. On day 0, bleomycin (5 mg/kg) was infused into the trachea to induce pulmonary injury and fibrosis. Briefly, the mice were anesthetized by continuous inhalation of diethyl ether, their airways were kept in a straight and horizontal state by placing their backs against a circular water bottle with their stomachs facing up. Then a laryngoscope was used to open their mouth to expose the glottis and a micro-sprayer (HRH-MAG4, Huironghe, China) was used to spray bleomycin into the lungs of mice through the glottis and tracheal. After bleomycin inhalation, the mice were kept upright, allowing bleomycin to enter as much of their lung tissue as possible by the gravity. On day 7, 1 × 10^6^ cells (BCs or MSCs) were resuspended with 50 μL PBS and then transplanted into lung tissue of anesthetized mice through endotracheal intubation. The cells of CoQ10 + BCs group were incubated with 10 mM CoQ10 for 24 h before transplantation and resuspended into 50 μL PBS containing 10 mM CoQ10, and the other steps were the same. The body weight of mice was recorded on day 7, 14 and 21 respectively. Lung tissues were extracted on day 21.

### Histological examination of lung tissue

The lungs were perfused with 4% paraformaldehyde overnight at 4 °C and performed standard paraffin embedding protocols containing dehydrated with gradient ethanol and embedded with wax. Slicer (Leica, Germany) was used to cut the lung tissues into slices with a thickness of 5–7 μm. The slices were placed on a slide coated by poly-lysine and then stored at room temperature until further use. H&E staining and Masson staining were performed according to the standard protocols. The degree of pulmonary fibers was evaluated through Ashcroft scoring standard. The blue part of Masson staining was quantified by Image J (collagen fibers).

### Measurement of lung hydroxyproline

To evaluate the extent of pulmonary fibrosis, the total collagen contents were measured using a hydroxyproline assay kit (abcam, Cat.ab222941) according to the manufacturer’s instructions. Briefly, the left lobes of lungs were completely dried in the oven and the weights of dry lobes were measured. The samples were then hydrolyzed at 95 °C in each tube containing 6N HCl for 2 days. The hydrolysates were centrifuged and then supernatants in each tube were transferred to fresh tubes. 5 μL of each sample or standard was mixed with 5 μL of citrate-acetate buffer into 96-wells plate in triplicate. 100 μL of Chloramine-T solution was added into each well and the mixture was incubated for 20 min. Then, 100 μL of Ehrlich’s solution was added into each well and the samples were incubated at 65 °C for 20 min. Absorbance was measured at 550 nm and the amount (μg/mg) of hydroxyproline was calculated by comparison to the standard curve.

### Immunofluorescence staining of pulmonary α-SMA, SPC, and human mitochondrial protein

The sections were reconstituted in citric acid buffer (Boster Biological Technology Co., ltd, California, USA) at 120 °C. The nonspecific antigens were blocked with 1% BSA for 20 min. Sections were incubated with primary antibody of α-SMA (SAB, Cat. 40482, 1:200 dilution,), or with primary antibody of SPC (Abcam, Cat. ab211326, 1:200) and anti-human mitochondria antibody (CHEMICON, Cat. MAB1273, 1:200) overnight at 4 °C, and then washed in PBS 3 times. Then sections were then incubated with the suitable secondary antibodies in dilution of 1:400 at room temperature for 1 h. Sections were washed in PBS 3 times and then incubated with DAPI (Beijing Solarbio Co., Ltd., Beijing, China) for 5 min, followed by washed in PBS 5 times. Slides were stored in a dark place at 4 °C, and staining of pulmonary α-SMA, SPC or human mitochondrial protein were recorded with a fluorescence microscope.

### Statistical analysis

The data were analyzed by Graphpad Prism 8.0 software (La Jolla, CA, USA). The survival curve was drawn using the Kaplan Meier method. All data were expressed as mean ± standard deviation (SD). T-test was used for analysis between two independent sample groups and single-factor analysis was used for comparison between multiple groups of data. *P* < 0.05 was considered statistically significant.

## Results

### Identification of BCs

**Fig. 1:**
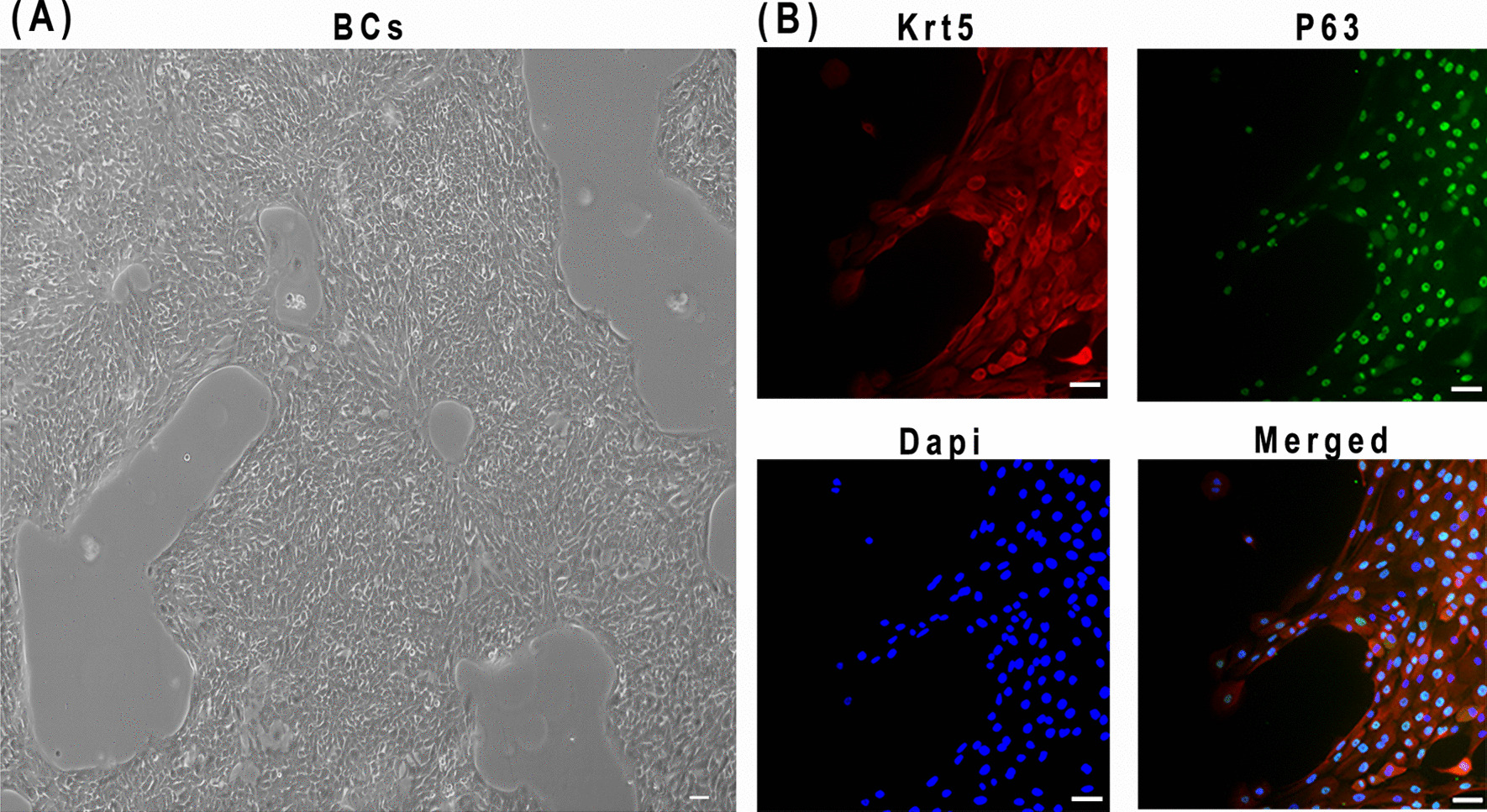
Culture and identification of BCs. BCs were primarily isolated and cultured from airway mucosal biopsy samples from non-fibrotic patients, with DMEM/F12 medium supplement with Y-27632, A-83-01, DMH-1, and CHIR99021. **A** The cultured BCs were observed under a light microscope. **B** BCs was stained by two cell makers, P63 (Green) and KRT5 (Red). DAPI was used for nuclear staining of BCs. Scale bars: 50 μm

Previous studies demonstrated that KRT5 and P63 were two characteristic markers of BCs. In the present study, expressions of KRT5 and P63 in BCs were stained by immunofluorescence staining (Fig. 1). Results showed that BCs grew in groups without dendritic or wedge-shaped margins, which displayed morphological characteristics of multipotent stem cells in the no-matrilgel culture system (Fig. 1A). KRT5 was mainly expressed in the cell membrane and cytoplasm, while P63 was mainly expressed in the nucleus of BCs (Fig. 1B). Most cells have KRT5 and P63 double-positive staining, indicating that the BCS cells cultured in the present study had stable and consistent phenotypes. BCs during P3 to P6 were used in subsequent experiments.

### Incubation with CoQ10 reduces H_2_O_2_-induced production of ROS in BCs

**Fig. 2:**
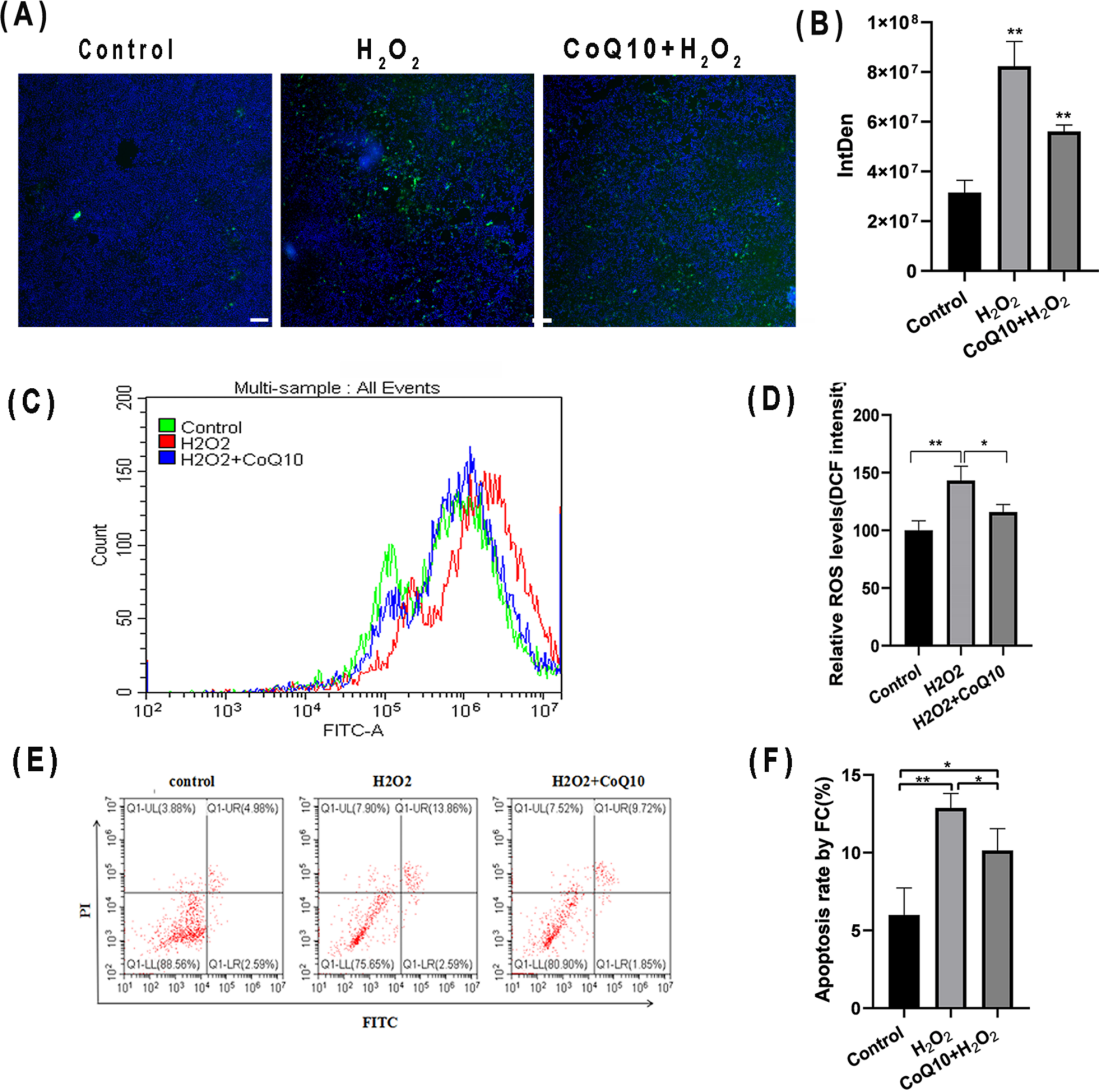
CoQ10 inhibits BCs apoptosis and increased ROS level in BCs causing by H_2_O_2_. **A**, **B** The cells of each group were stained with DCFH-DA reactive oxygen fluorescent probe. Fluorescence representative pictures of cells in the blank group, H_2_O_2_ group and CoQ10 + H_2_O_2_ group were taken with a fluorescence microscope, Scale bars: 200 μm. **C**, **D** The cells of each group were stained with DCFH-DA reactive oxygen species fluorescent probe, and the ROS levels of cells in the blank group, H_2_O_2_ group and CoQ10 + H_2_O_2_ group were detected by flow cytometry. **E**, **F** The cells of each group were stained with Annexin-FITC/PI dye, and the apoptotic number of cells in the blank group, H_2_O_2_ group and CoQ10 + H_2_O_2_ group was detected by flow cytometry. *P < 0.05, **P < 0.01, *ns* no significant

The stained BCs were photographed by a fluorescence microscope DCFH-DA. Results showed that the fluorescence intensity of ROS was significantly increased after incubation with 1 mM H_2_O_2_ for 6 h, which was remarkably reduced by CoQ10 pretreatment (Fig. 2A, B). Moreover, intracellular ROS production in BCs after CoQ10 and H_2_O_2_ pretreatment was recorded by flow cytometry (Fig. 2C). Results showed that the number of ROS-positive cells in the H_2_O_2_ group increased significantly than that in the normal control group, which was remarkably blocked by pretreatment with CoQ10 (Fig. 2D).

### Incubation with CoQ10 reduces H_2_O_2_-induced apoptosis of BCs

H_2_O_2_-induced apoptosis and necrosis of BCs were measured by flow cytometry (Fig. 2E, F). The proportion of BCs undergoing apoptosis and necrosis in the H_2_O_2_ group increased to 24.35%, as compared with the proportion of apoptotic and necrotic BCs was 11.45% in the normal control group. Moreover, the proportion of BCs undergoing apoptosis and necrosis in the CoQ10 + H_2_O_2_ group (19.09%) was decreased significantly as compared to that in the H_2_O_2_ group, indicating that CoQ10 pretreatment reduced H_2_O_2_-induced apoptosis of BCs.

### Transplanting BCs combined with CoQ10 alleviated the symptoms of bleomycin-induced IPF in C57/B6 mice

**Fig. 3:**
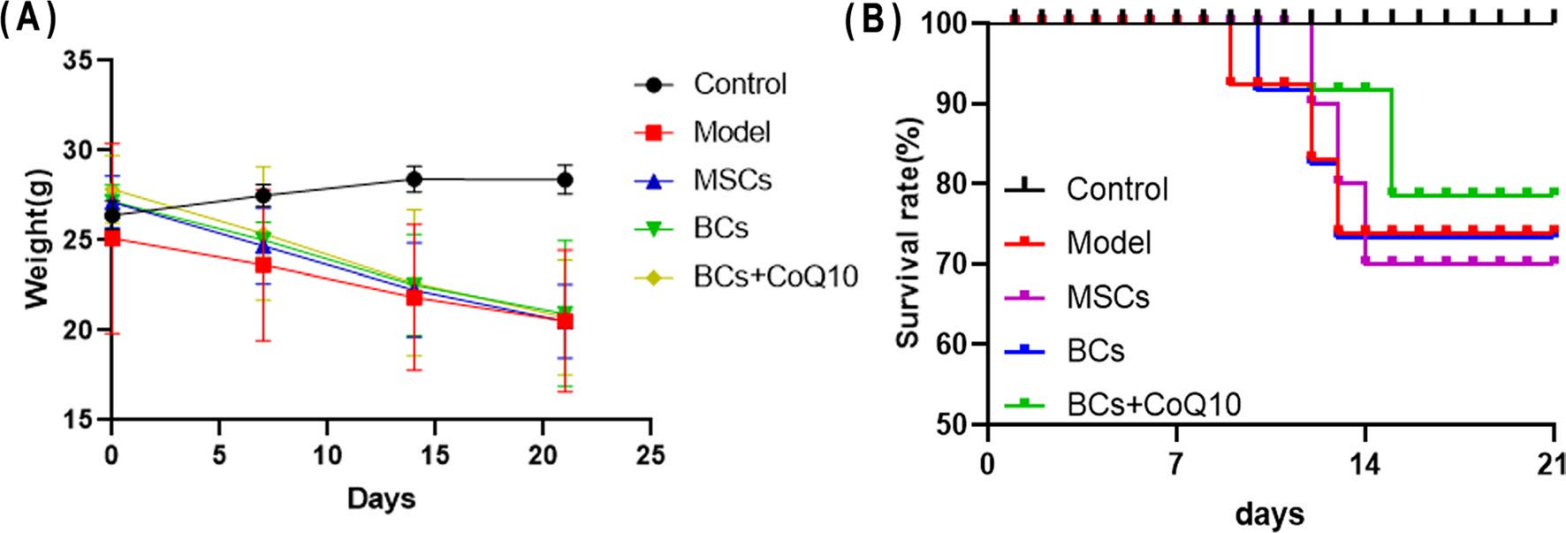
Changes in body weight of mice and survival curves after bleomycin instillation and cells transplantation. **A** Changes in body weight of mice in each group after bleomycin exposure. **B** The survival rate of mice in each group. The mortality of the mice during 21-day observation period was 30% (3/10), 30% (3/10), 30% (3/10), 20% (2/10), 0% (0/10) for the Model group, MSC group, BCs group, BCs + CoQ10 group, control group, respectively

After mice were infused with bleomycin (5 mg/kg, in 50 μL PBS) into the trachea to induce pulmonary injury and fibrosis, cells (BCs or MSCs) were transplanted into lung tissue of anesthetized mice through endotracheal intubation on day 7. The changes in bodyweight of mice during the modeling period were recorded (Fig. 3A). Results showed that the bodyweight of mice in the normal control group exhibited increase during the modeling period. However, the bodyweight of all animals with bleomycin instillation, including in the model group and three groups with cells transplantation, was gradually decreased with the prolongation of molding time. Among them, the mortality of mice in the CoQ10 + BCs group was 20%, while it was 30% in the BCs group at the endpoint of modeling (on day 21), but the mice in the normal control group did not die during whole 3 weeks (Fig. 3B).

**Fig. 4:**
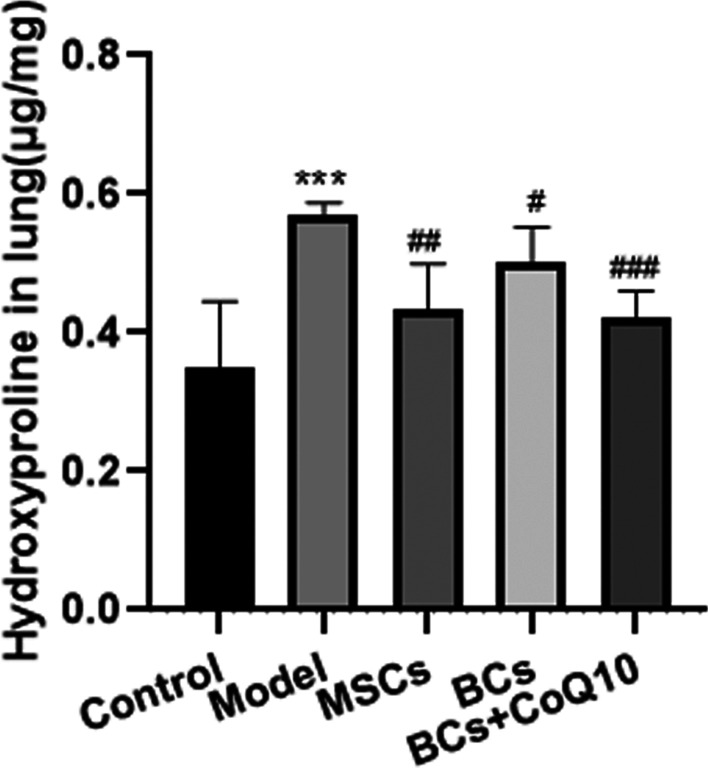
The HYP content in mice lung tissue after bleomycin instillation and cells transplantation. The collagen production in the lung tissue of mice was evaluated by hydroxyproline (HYP) assay kit. the amount of HYP (μg/mg) was calculated by comparison to the standard curve. Data was represented as the mean ± standard deviation (SD), n = 6 per group. ***P < 0.001 vs. control group; ^#^*P* < 0.05 vs. model group; ^##^*P* < 0.01 vs. model group; ^###^P < 0.001 vs. model group

The hydroxyproline (HYP) content in lung tissue of model group mice was increased remarkably to 1.52-folds as compared to that in normal control group animals. Moreover, bleomycin-induced increase in HYP was significantly inhibited after transplantation of MSCs, BCs alone or combined with CoQ10 (Fig. 4A).

**Fig. 5:**
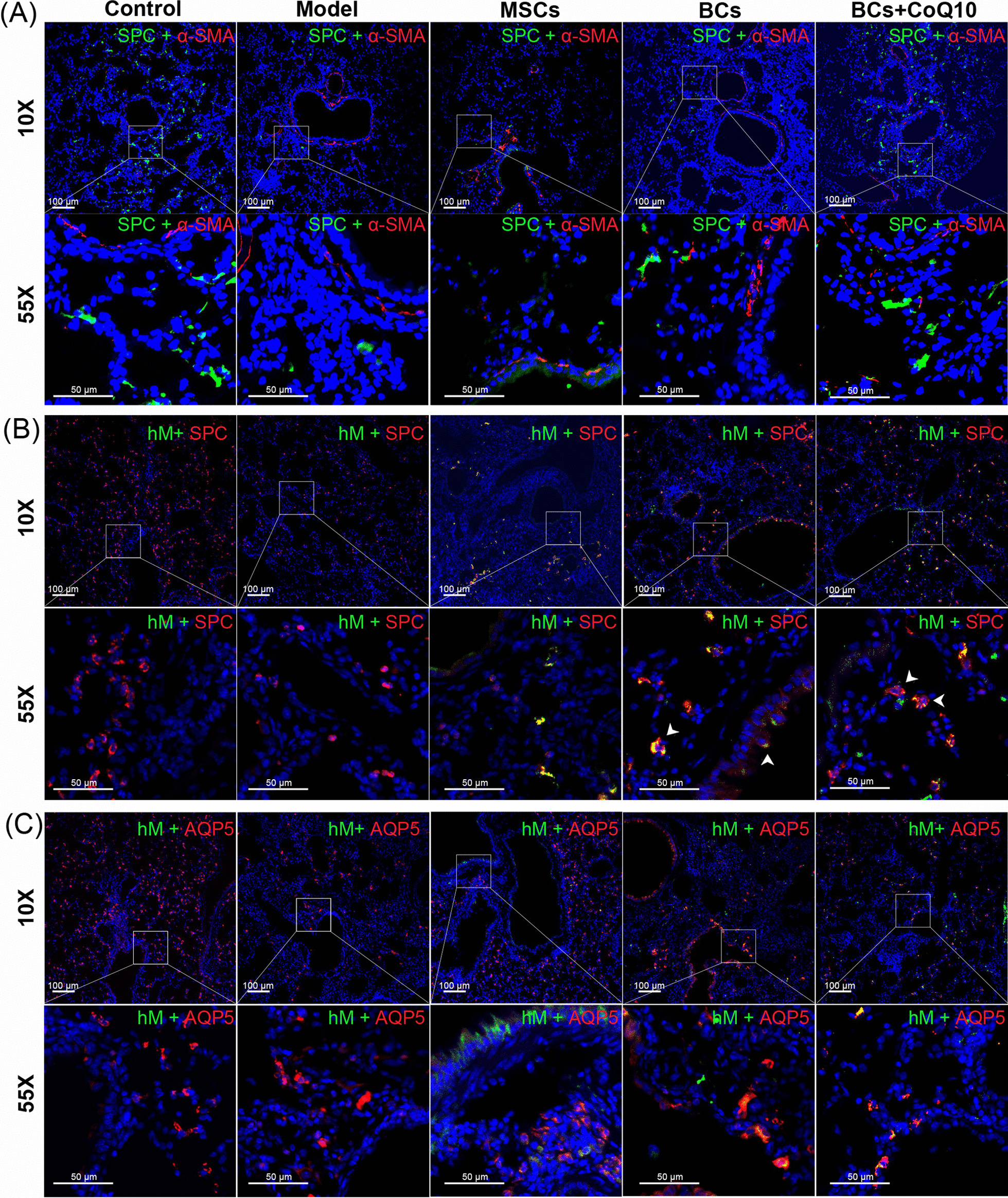
Immunofluorescence of α-SMA, SPC, AQP5, and hM in the lung section of each group after bleomycin instillation and cells transplantation. The right lobes of lungs were cut into slices with a thickness of 5–7 μm, incubated with suitable antibodies for IFC staining. **A** Anti-α-SMA antibody (red), anti-SPC antibody (green), and DAPI (blue) staining showed the degree of fibrosis and expression of SPC in the lung tissues of mice. **B** Staining with antibodies of SPC (red) and human mitochondria (hM, green) that showing survival of MSCs or BCs, and differentiation into type II alveolar epithelial cells in the lung tissues of mice. **C** Staining with antibodies of AQP5 (red) and hM (green) that showing survival of MSCs or BCs, and differentiation into type I alveolar epithelial cells in the lung tissues of mice. Mouse pulmonary epithelial cells only shows green fluorescence in cytoplasm, while undifferentiated human MSCs or BCs with only red fluorescence, and differentiated MSCs or BCs displays overlapping positive staining of green and red fluorescence (white arrow). Scale bars: 100 μm. n = 6 per group

In order to test whether the transplanted MSCs or BCs could survive and differentiate into alveolar epithelial cells in lung tissue of mice, antibodies of SPC, AQP5, α-SMA, and hM were applied to display the transplanted cells located in lung tissue and the severity of fibrosis (Fig. 5). Results showed that the deposition of α-SMA was increased remarkably after modeling by bleomycin instillation, which was significantly alleviated by transplantation of MSCs, BCs alone, or combined with CoQ10. Moreover, bleomycin instillation could decrease the positive staining of SPC remarkably in lung tissue of model group mice, of which was reversed by BCs transplantation, especially in combination with CoQ10 (Fig. 5A). The double staining of SPC and hM showed that SPC was uniformly expressed in alveolar epithelial cells of control group mice. After bleomycin instillation, SPC expression in the lung tissues of mice was decreased and became very uneven, suggesting that the alveolar epithelial cells were damaged after bleomycin instillation. Moreover, a small number of transplanted MSCs showing green fluorescence of hM could be detected in lung tissue of MSCs group mice, indicating that the transplanted MSCs were survived in mouse lung. However, SPC was rarely expressed in transplanted MSCs, suggesting that these cells did not differentiate in mice lung. Some transplanted BCs in both BCs group and BCs + CoQ10 group showed overlapping staining of red fluorescence of SPC and green fluorescence of hM, indicating that these transplanted BCs successfully differentiated into type II alveolar epithelial cells in the lung tissues of mice. In addition, some MSCs with hM positive staining were remained in the small airway epithelium of the MSCs group mice, while a few BCs with SPC and hM overlapping positive staining were observed in the small airway epithelium of the BCs group mice, suggesting that some MSCs and BCs were remained in the airway of the two groups of mice. However, residual BCs was rarely found in the small airway of lung tissue in BCs + CoQ10 group (Fig. 5B). The double staining of AQP5 and hM showed that AQP5 was widely distributed in lung tissue of healthy mice, which was significantly decreased after bleomycin instillation, especially in the area adjacent bronchiole. Moreover, the positive staining of hM could be observed in lung tissues of mice transplanted with MSCs and BCs, and almost no cells showed overlapping staining of AQP5 and hM, suggesting that MSCs and BCs couldn’t differentiate into type I alveolar epithelial cells after transplantation (Fig. 5C).

**Fig. 6:**
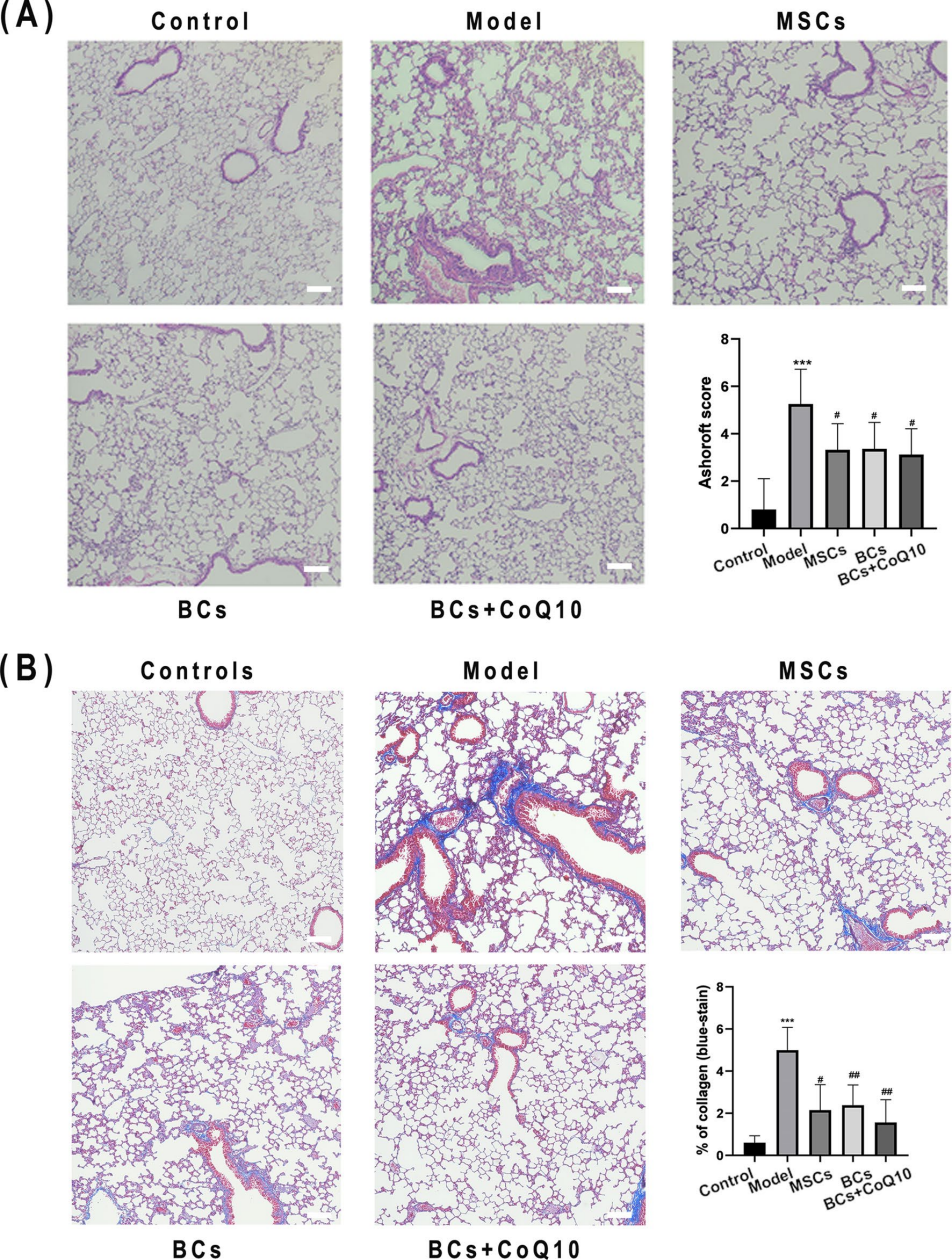
Histopathological staining of the lung tissues after bleomycin instillation and cells transplantation. Pulmonary tissue sections were prepared by right lobes of lungs on day 21 and stained with H&E (**A**) and Masson’s trichrome staining (**B**). H&E staining showed the infiltration of inflammatory cells, and lung epithelial cells injury and repair disorder. Masson’s trichrome staining visualized collagen deposition in lung section of mice. Scale bars: 100 μm; ***P < 0.001 vs. control group; ^#^*P* < 0.05 vs. model group; ^##^*P* < 0.01 vs. model group. n = 6 per group

The H&E staining pictures showed that rare exudation in the alveolar cavity and inflammatory cells infiltration was founded in the lung tissue of mice in normal control group, while the lung tissue structure of the model group mice was completely destroyed (Fig. 6A). In the bleomycin-induced mice, the lung intervals were significantly thickened, the capillary morphology was unobvious and the alveolar structures were extremely difficult to be distinguished. Also, a large amount of fibrous exudate was deposited, and some regions of the lung tissue were consolidated. On the contrary, the exudation degree in the alveolar cavity and inflammatory cells infiltration in three kinds of cell transplantation groups were significantly weakened and the lesions were also reduced.

In Masson staining sections as shown in Fig. 6B, there was no fibrous tissue hyperplasia in the lung tissue of normal control group animals. However, collagen fibers that dyed blue were significantly increased around small bronchus and lung interstitium in bleomycin-injured model group animals, especially distributed in the basement membrane of bronchial branches and severely damaged bronchus. On the contrary, collagen fibers deposition was significantly reduced in all three cell transplantation groups, especially in the BCs + CoQ10 group animals (*P* < 0.01).

## Discussion

Accumulating evidence suggested that stem cells-based therapies may be used for lung regeneration and modulation of inflammatory and fibrotic processes [5]. In the present study, we observed that administration of MSCs, BCs, or BCs combining with CoQ10 into lung tissue of mice 7 days after bleomycin instillation, ameliorated the histopathological characteristics of lung tissues and prevented pulmonary fibrosis development. Cells transplantation reduced the degree of pulmonary fibrosis as compared with the model group. Slices staining showed that both inflammatory cells infiltration and collagen fiber content in cells transplanted animals was significantly reduced as compared with the animals in the model group, as well as pulmonary α-SMA. The hydroxyproline content in the BCs + CoQ10 group was lower than that in the BCs group. Furthermore, our results showed that CoQ10 could inhibit H_2_O_2_-induced ROS production in BCs and cell apoptosis. CoQ10 played a protective role to BCs against oxidative stress and enhanced the differentiation of transplanted BCs, which indicates that CoQ10 may improve the survival rate of transplanted cells in the pulmonary microenvironment.

Previous studies reported that stem cells have differentiation potential and can differentiate into tissue-resident lung cells to repair pulmonary damage. Therefore, stem cells therapy is expected to become a radical therapy to delay or reverse IPF [30]. Among all cells with clinical potential, MSCs are easy to obtain and handle and with lowest immunogenicity [31]. However, it is widely recognized that transplanted MSCs perform repair function mainly through the paracrine or immunomodulatory mechanism, with no evidence showing that they can reconstitute lung structure for regeneration purposes [9]. Moreover, MSCs are now recognized as key players at distinct steps of tumorigenesis [32], transplantation of MSCs to treat IPF may bring unpredictable oncogenic risks. Adult stem cells are undifferentiated cells existing in a differentiated tissue that can self-proliferate, renew and can be directed to differentiate into mature functional cells of that tissue. Such cells are relatively easy to obtain and can be obtained directly from the patient, and then be cultured and proliferated in vitro. It avoids the immune rejection problems caused by allogeneic cells transplantation. Theoretically, the tumorigenic risk of adult stem cells is low, and there are few ethical controversies [33]. Hematopoietic stem cells are mature adult stem cells that are transplanted to treat hematological diseases [34], and limbal stem cells are also mature adult stem cells that are applied in clinical practice [35]. BCs are adult stem cells located in the lungs, and can also be differentiated from iPSCs [36]. Studies have shown that BCs can migrate to the damaged site after alveolar injury, differentiate into alveolar epithelial cells, and then repair the damage [21]. Shi et al. found that transplantation of BCs could alleviate bleomycin-induced IPF in mice [22]. Moreover, results in present study proved that the transplanted BCs could differentiate into functional alveolar epithelial cells to repair the damage.

Cotreatments by different factors and drugs can influence the biological activity of transplanted cells ex vivo and in vivo, thereby improving their reparative efficacy for applications in current regenerative medicine [37]. But some factors could activate fibroblasts, which worsening pulmonary fibrosis symptoms [38]. In the present study, CoQ10 was designed to improve the transplantation efficiency of BCs by making cells better adapt to the microenvironment with high oxidative stress levels. Cells were pretreated with CoQ10 before transplantation to improve cell survival signal. On the other hand, transplanting cells combining with CoQ10 alleviated oxidative stress levels in the microenvironment for the survival of cells after transplantation.

However, CoQ10 may also have a pro-differentiation effect which needs further verification, and the expression of cell survival signal after pretreatment needs further exploration. We cannot rule out that the role of BCs is to promote the differentiation of endogenous adult stem cells into alveolar epithelial cells through indirect paracrine, which is still an area that needs to be studied in more detail. At last, the survival rate of transplanted cells in each group also needs further verification.

## Conclusions

CoQ10 blocks H_2_O_2_-induced apoptosis of BCs and ROS production in vitro, and enhances the efficacy of BCs transplantation on bleomycin-induced IPF in mice.

## Data Availability

All the data obtained from the present study are available from the corresponding author under reasonable request.

## Abbreviations

IPF: Idiopathic pulmonary fibrosis
BC: Airway basal stem cells
MSCs: Mesenchymal stem cells
iPSCs: Induced pluripotent stem cells
CoQ10: Coenzyme Q10
HYP: Hydroxyproline
